# The Association of Tetrameric Acetylcholinesterase with ColQ Tail: A Block Normal Mode Analysis

**DOI:** 10.1371/journal.pcbi.0010062

**Published:** 2005-11-18

**Authors:** Deqiang Zhang, J. Andrew McCammon

**Affiliations:** 1 Howard Hughes Medical Institute, University of California, San Diego, California, United States of America; 2 Department of Chemistry and Biochemistry, University of California, San Diego, California, United States of America; 3 Center for Theoretical Biological Physics, University of California, San Diego, California, United States of America; 4 Department of Pharmacology, University of California, San Diego, California, United States of America; Weill Medical College of Cornell University, United States of America

## Abstract

Acetylcholinesterase (AChE) rapidly hydrolyzes acetylcholine in the neuromuscular junctions and other cholinergic synapses to terminate the neuronal signal. In physiological conditions, AChE exists as tetramers associated with the proline-rich attachment domain (PRAD) of either collagen-like Q subunit (ColQ) or proline-rich membrane-anchoring protein. Crystallographic studies have revealed that different tetramer forms may be present, and it is not clear whether one or both are relevant under physiological conditions. Recently, the crystal structure of the tryptophan amphiphilic tetramerization (WAT) domain of AChE associated with PRAD ([WAT]_4_PRAD), which mimics the interface between ColQ and AChE tetramer, became available. In this study we built a complete tetrameric mouse [AChE_T_]_4_–ColQ atomic structure model, based on the crystal structure of the [WAT]_4_PRAD complex. The structure was optimized using energy minimization. Block normal mode analysis was done to investigate the low-frequency motions of the complex and to correlate the structure model with the two known crystal structures of AChE tetramer. Significant low-frequency motions among the catalytic domains of the four AChE subunits were observed, while the [WAT]_4_PRAD part held the complex together. Normal mode involvement analysis revealed that the two lowest frequency modes were primarily involved in the conformational changes leading to the two crystal structures. The first 30 normal modes can account for more than 75% of the conformational changes in both cases. The evidence further supports the idea of a flexible tetramer model for AChE. This model can be used to study the implications of the association of AChE with ColQ.

## Synopsis

Acetylcholinesterase (AChE) breaks down acetylcholine in the neuromuscular junction and other cholinergic synapses to terminate neuronal signals. AChE exists as tetramers anchored by structural subunits to the cell membranes in the brain or the basal lamina in the neuromuscular junction. Based on a crystal structure of the tetramerization domain of AChE with a proline-rich attachment domain of the anchoring proteins, a symmetric model of the complex of AChE tetramer with the anchoring protein tail was constructed. Block normal mode analysis revealed the presence of several low-frequency, low-barrier normal modes corresponding to inter-subunit motions. Previous crystal structures of AChE tetramer could be rationalized using these normal modes. These low-frequency modes are due to the presence of a flexible hinge in the structure of AChE. This study paints a picture of a flexible AChE tetramer with different conformational states interconverting easily under physiological conditions, which has important implications on the function of AChE. In particular, AChE is not trapped in the compact tetramer structure, for which access of substrate to two of the active sites is somewhat limited. Rather, the tetramer fluctuates to expose all four of its active sites to ensure rapid removal of acetylcholine.

## Introduction

Acetylcholinesterase (AChE; E.C. 3.1.1.7) rapidly hydrolyzes acetylcholine to terminate neurotransmissions at cholinergic synapses [1,2]. The reaction is very fast, approaching the diffusion limit. AChE has three different molecular forms due to an alternate splicing scheme at the C-terminus [3]. The T-subtype (AChE_T_) with a 40-residue C-terminal “t-peptide” is the only form expressed in the brain and adult muscles of normal adult mammals [4]. In vertebrate cholinergic synapses, tetramers of AChE_T_ are associated with either collagen-like Q subunit (ColQ) or transmembrane proline-rich membrane-anchoring protein (PRiMA) [5,6]. ColQ is a structural protein that anchors AChE_T_ to the synaptic basal lamina [5,7], and PRiMA is a membrane protein that anchors AChE_T_ to the membrane of neuronal synapses in the brain [6]. They both contain a proline-rich attachment domain (PRAD) near the N-terminus, which is the site for interacting with the t-peptide of AChE. The PRAD has three and five consecutive proline residues, and it has been shown that synthetic polyproline could replace PRAD in its association with AChE_T_ tetramers [8]. In AChE_T_ the t-peptide is absolutely required in its association with PRAD [9]. The sequence of the t-peptide is highly conserved throughout vertebrates, with a cysteine at −4 position from the C-terminus and a series of seven aromatic residues, including four equally spaced tryptophans. Because the t-peptide constitutes an autonomous interacting domain, it has been named “the tryptophan amphiphilic tetramerization” (WAT) domain. In this notation, AChE_T_ is equivalent to AChE + WAT [10].

Recently the crystal structure of PRAD/WAT complex was solved at 2.35 Å resolution [11]. The complex has the expected [WAT]_4_PRAD stoichiometry. Four parallel α-helical WAT chains wrap around a single antiparallel PRAD helix, which itself has a left-handed polyproline II conformation. Each WAT helix assumes a coiled-coil conformation, and all four of them form a left-handed supercoil around the PRAD (Figure 1A). The WWW motif in the WAT makes repetitive hydrophobic stacking and hydrogen bond interactions with the PRAD. The four WAT chains are related by a 4-fold screw axis around the PRAD. The strength of PRAD–WAT interaction is very tight, with no monomer of WAT detected in the range of 10^−10^ to 10^−12^ M [8].

**Figure 1:**
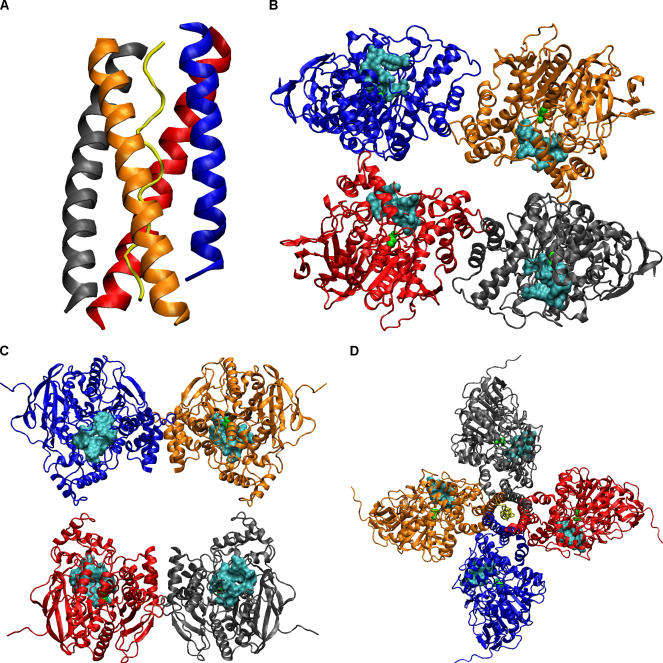
The Structures Related to AChE Tetramerization (A) The [WAT]_4_PRAD complex structure. (B) The compact tetramer structure. (C) The loose tetramer structure. (D) The [AChE_T_]_4_–ColQ complex model constructed according to the [WAT]_4_PRAD complex structure. Each chain is colored differently (A, blue; B, red; C, gray; D, orange; ColQ, yellow). The catalytic S203 was shown as a green ball model for each AChE subunit, and the cyan surfaces are residues near the peripheral site.

It remains unknown how the four AChE subunits are arranged in the tetramer associated with the PRAD. Low-resolution crystallographic studies revealed two distinct three-dimensional structures of AChE tetramer [12]. Both crystal structures show a dimer of dimers, i.e., there is no 4-fold symmetry to relate all four subunits. In one structure two AChE dimers are close with all four C-terminal sequences aligning to the same direction (referred to as the compact tetramer; Figure 1B), and in the other structure the space between the two dimers is large and the four C-terminal sequences are aligned antiparallel to the middle (referred to as the loose tetramer; Figure 1C). The crystals were grown from trypsin-digested, collagen-tailed AChE and should both have WAT and PRAD preserved; although electronic densities were seen, it was not possible to resolve them. It was suggested that the flexibility of AChE tetramers might be related to the regulation of catalysis [2,12]. To test this, reaction-rate calculations were conducted using these two tetramer structures and a morphed intermediate structure. The results showed that the rate per active site was reduced due to active site occlusion and sink–sink competition compared to the monomeric form, but could be partly compensated by electrostatic enhancements in the tetramers [13]. The rate reduction due to active site occlusion was particularly notable for the compact tetramer [13].

Efforts to combine the PRAD/WAT structure and the two AChE tetramer structures were not very successful. The problem was that in both tetramer structures the four AChE subunits lacked 4-fold symmetry as seen in the [WAT]_4_PRAD complex crystal structure [11]. Since the four WAT chains are staggered in the structure, it is impossible, without substantial distortion of the [WAT]_4_PRAD complex and/or the AChE tetramer structures, to dock the [WAT]_4_PRAD complex along the 2-fold axis between the pairs of AChE dimers. In fact, the superhelical axis of the [WAT]_4_PRAD complex structure is at an angle of about 30° to this 2-fold axis [11].

Considering the tight association between PRAD and WAT, the weak affinity of the AChE dimer, and the limited contact between the two dimers in the tetramer, it is reasonable to assume that the PRAD–WAT interaction dominates over the inter-subunit interaction in the association of AChE tetramer with ColQ. In fact, AChE only exists as a soluble monomer if the WAT sequence is deleted [9], indicating that the dimerization forces are weak. In a previous study of subunit association in cholinesterases, this interaction was identified as the weak hydrophobic interaction, compared with the strong interaction seen between WAT and PRAD [14]. It has also been shown that if two proteins, for example, AChE and alkaline phosphatase—both with WAT sequences at their C-termini—are mixed with PRAD at various ratios, tetramers containing three subunits of one protein and one of the other are underrepresented [10]. This points to a symmetric [AChE_T_]_4_–ColQ complex form in physiological conditions. Since the two crystal structures of AChE tetramer were generated experimentally, they have to be accessible from the physiological model through conformational changes.

In this study, we built an [AChE_T_]_4_–ColQ complex model strictly following the PRAD–WAT interaction. Although molecular dynamics (MD) is a useful tool to observe dynamics in conformational changes, the size of the system (more than 2,300 residues) poses too much a challenge in this case [15]. Block normal mode analysis (BNMA) uses coarse-grained representation for the protein to reduce the computational cost and has been shown to be a useful tool in predicting low-frequency motions seen in large protein assemblies, for example, the swelling of a virus capsid [16–18], the ratchet motion of the ribosome [19], the collective motions in HIV-1 transcriptase [20], myosin [21,22], ATPase [22,23], and chaperonin GroEL [24], etc. These low-frequency motions are often related to the conformational changes required by the biological functions of the protein assemblies.

Here we applied BNMA to the [AChE_T_]_4_–ColQ complex model and calculated the 100 lowest normal modes. By projecting the conformational changes onto these normal modes, it was found that the two AChE tetramer crystal structures could be rationalized by using these low-frequency normal modes.

## Results/Discussion

Our [AChE_T_]_4_–ColQ complex model has a quasi-4-fold axis as shown in Figure 1D. The bending angle between the WAT helix and the C-terminal helix of AChE is approximately 45°. Structural relaxation changed the model by 0.95 Å in root mean square deviation (RMSD) of the backbone atoms calculated by the ptraj module of Amber8. If viewed from the presynaptic side (i.e., the WAT sequence on the top), each gorge to the active site is located at the clockwise side of AChE and orients almost exactly parallel to the tetramer plane. In this model each AChE subunit has its own quarter of space for attracting substrate, and no active site is blocked by adjacent subunits. Presumably the sink–sink competition is less in such an arrangement, because the average sink–sink distance is larger than that in the two crystal structures of the tetramer. These two factors can both increase the diffusion-controlled reaction rate for the [AChE_T_]_4_–ColQ complex. Reaction-rate calculations are currently underway using this model.

A simplified BNMA was done to calculate the low-frequency modes, using the [AChE_T_]_4_–ColQ structure model. The first 100 lowest frequency normal modes were obtained. These normal modes are sufficient to capture all the collective motions, and motions with higher frequency are usually localized to a small domain [21,25]. Figure 2 shows the root mean square fluctuations (RMSFs) derived from Equation 1. It appears that the largest structural fluctuations are located at the WAT and PRAD sequences. This is consistent with the fact that these sequences in the two crystal structures (1C2O and 1C2B) of the AChE tetramer were disordered. As can be seen in the correlation map of motions discussed below, the motion experienced by this region is rigid-body in nature. Therefore it does not contradict our hypothesis that WAT and PRAD have very high affinity. Other regions showing high fluctuation are residues 380 to 390 and residues 265 to 276. They are both loops connecting α helices. Residues 380 and 390 are located at the interface between the catalytic domain of AChE and ColQ; therefore the large fluctuation indicates that there is no strong association between them at this region.

**Figure 2:**
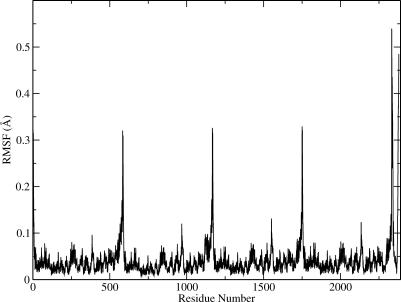
The RMSF of the [AChE_T_]_4_–ColQ Complex as Calculated from the 100 Lowest Frequency Modes All residues are numbered continuously (chain A: 1–583, B: 584-1166, C: 1167–1749, D: 1750–2332, ColQ: 2333–2379).

The correlation map can identify collective motions, which are often important large-scale motions related to the protein's biological function. Due to the large amount of data, only one AChE_T_ subunit and the ColQ are presented in the motion correlation map in Figure 3. The WAT domain, which corresponds to residues 544 to 583 in Figure 3, has little or no correlation with the catalytic domain of AChE. Instead, it moves together with ColQ (residues 584 to 630), as implied by the high correlation between the WAT and ColQ. This further demonstrates that the interaction between AChE and ColQ is weak, and the interaction between WAT and ColQ is strong. An interesting region in the correlation map can be found for residues 355 to 410. These residues form an α helix bundle themselves and are isolated in the correlation map from the rest of the catalytic domain of AChE. Therefore it appears that these residues form a subdomain. Structurally, Pro410 breaks this subdomain from a long helix, and Ser355 is connected to a flexible loop. They can be considered as hinges connecting different domains. We note that Pro410 is almost universally conserved in all cholinesterases. In our [AChE_T_]_4_–ColQ complex model, this subdomain makes contact with the C-terminal extension of ColQ, as is evident from the correlation map. In addition, this subdomain participates in the inter-subunit interface with the clockwise neighboring subunit.

**Figure 3:**
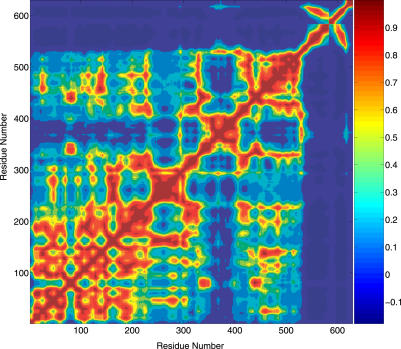
The Motion Correlation Map of the [AChE_T_]_4_–ColQ Complex as Predicted by BNMA Only one AChE subunit and ColQ were plotted here (AChE: 1–583, ColQ: 584–630).

Conformational changes in proteins can be considered as linear combinations of displacements along low-frequency normal modes. Since the two crystal structures of AChE tetramer were real snapshots of the [AChE_T_]_4_–ColQ complex, they represent two conformational states with large conformational changes to the complex structure. By projecting the conformational changes onto the eigenvector of each normal mode, it is possible to identify the degree to which each mode is involved in the conformational change [22].

Figure 4 shows the results for the involvement analysis of these conformational changes. In the case of the compact tetramer, the lowest frequency mode (except the trivial modes corresponding to translation and rotation) has the highest coefficient at 0.53 as seen in Figure 4A. Such a large involvement coefficient indicates that this mode is highly relevant to the conformational change from the symmetric model to the compact tetramer structure. Figure 5A illustrates the collective motions in this mode by plotting the displacement onto each residue. The frequency of this mode is 1.39 cm^−1^. It seems that in this mode the subunits move closer to each other, as the inter-subunit distance is shorter in the compact tetramer structure compared to the symmetric model of [AChE_T_]_4_–ColQ complex. The cumulative involvement plot in Figure 4C shows that the first 30 lowest frequency modes can account for 80% of the motions in the conformational change to the compact tetramer structure.

**Figure 4:**
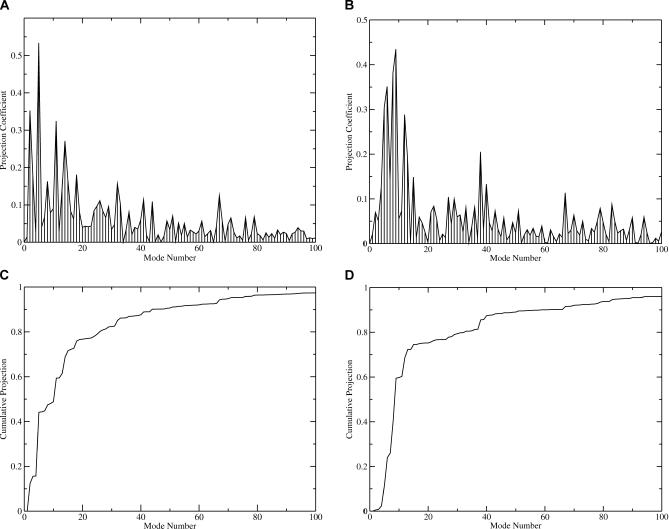
The Involvement Analysis of the Low-Frequency Modes of the [AChE_T_]_4_–ColQ Complex The involvement of the modes was shown for the conformational change to (A) the compact and (B) loose tetramer structures. The cumulative involvement of the modes was shown for (C) the compact and (D) loose tetramer structures.

**Figure 5:**
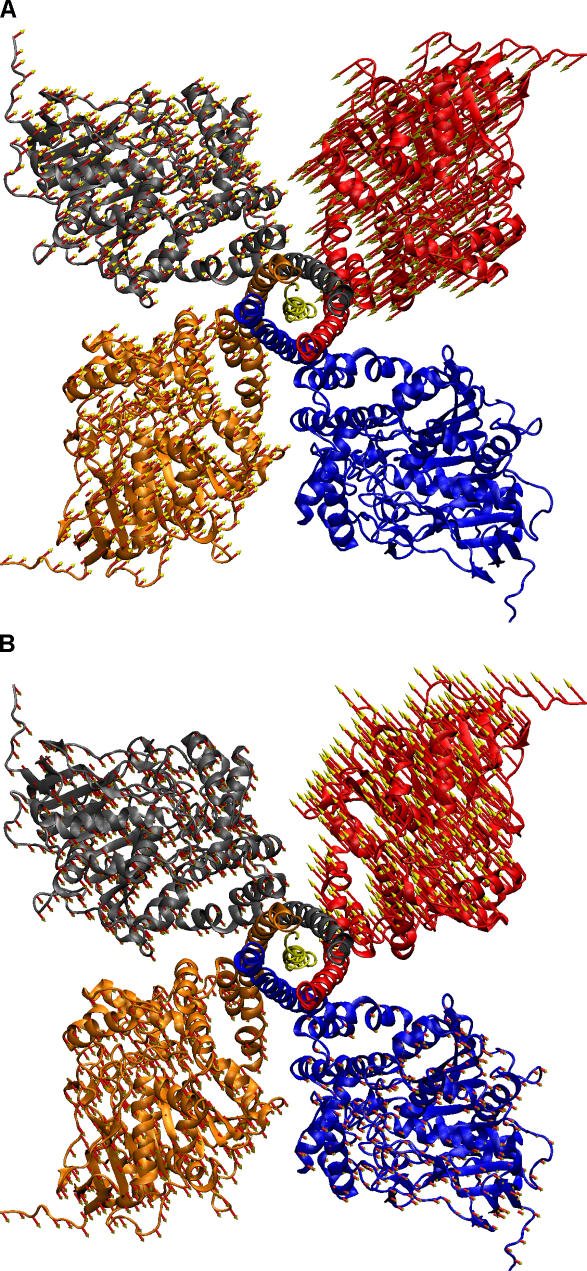
Illustration of Motions in the Two Lowest Frequency Modes from BNMA The arrow represents the amplitude and direction of the displacement experienced by each residue in the conformational change. Frequencies of the two modes are (A) 1.49 cm^−1^ and (B) 1.66 cm^−1^.

In the case of the loose tetramer structure, the most involved modes are the second and fourth lowest frequency modes with involvement coefficients of 0.35 and 0.44, respectively. The vibrational frequencies of these two modes are 1.66 cm^−1^ and 1.89 cm^−1^, respectively. Figure 5B illustrates the second lowest frequency mode by plotting displacements onto each residue. In this mode, one AChE subunit moves away from others, thus increasing the inter-subunit distance. In the fourth lowest frequency mode, one subunit rotates relative to the others (not shown in Figure 5). This motion can change the relative orientation of the gorges, as the gorge openings are in an angle in the crystal structures, while they are all in a plane in our model. In fact, all first seven lowest frequency modes are collective inter-subunit motions involving the four AChE catalytic domains, with little or no involvement from the WAT domain or ColQ. The first 30 modes can account for 75% of the motion in the conformational change in the loose tetramer as seen in Figure 4D.

All the seven inter-subunit motions involve a flexible hinge in residues 540 to 545 (D544 is the first residue in the WAT sequence), with T543 at the center of the flexible hinge. These residues have no correlation in motion with either AChE or the WAT/PRAD complex as seen in Figure 3. Visualization of each individual normal mode confirmed these hinge residues. This is in excellent agreement with secondary structure prediction that these residues form a loop and with the experimental finding that cysteins introduced in this region can efficiently form homomeric disufide bonds in dimers [26]. The conformational flexibility of these hinge residues allows WAT and AChE to each maintain their secondary structures. If these residues were deleted or made more rigid, it would affect the tetrameric association of AChE with PRAD as the secondary structure of either AChE or WAT has to be broken at the joint.

Using a simplified linear combination of these 100 normal modes, we generated two series of transient models of the [AChE_T_]_4_–ColQ complex that minimize the RMSD to the two crystal structures. The RMSD between the models of the [AChE_T_]_4_–ColQ complex and the compact tetramer structure decreased from 23.9 Å to 15.7 Å, while for the loose tetramer structure it went from 28.9 Å to 17.6 Å. The large RMSD values are due to the large translational and rotational movement required for each AChE subunit to realign between the [AChE_T_]_4_–ColQ complex model and the crystal structures. As a comparison, the RMSD for the two crystal structures is 34.9 Å, i.e., even larger than the initial RMSD between the [AChE_T_]_4_–ColQ complex model and the two crystal structures. When the two final models of the [AChE_T_]_4_–ColQ complex from projection were examined, it was found that they were significantly distorted in respect to the monomeric AChE. However, both models show a similar dimer of dimers as seen in the two crystal structures. The orientations of the active site gorges were originally all parallel to the tetramer plane. After applying projected motions from BNMA, two gorges were pointing downward and the other two slightly upward. It should be noted that this is a rather approximate demonstration using a simplified linear combination of normal mode motions. The application of normal modes for proteins is strictly only valid for very small conformational motions.

Normal mode analysis (NMA) can tell us what intrinsic low-frequency or large-scale motions the molecule or molecular assembly has. Conformational changes can be achieved by activating these modes to cross low-energy barriers by ligand binding or environmental changes. As indicated by the low-frequency inter-subunit normal modes, each AChE subunit in the [AChE_T_]_4_–ColQ complex may possess significant flexibility due to the presence of a flexible hinge at the interface of AChE and WAT. Under physiological conditions, there is a distribution of many different conformational states, including the two crystal structures and the symmetric tetramer model we constructed based on the [WAT]_4_PRAD complex structure. Due to the low barrier uncovered by our analyses, these conformational states can easily interconvert. Experimental findings support such a conformational transition. For example, under conditions that the compact AChE tetramer is grown, all four active site gorges can be occupied by fasciculin [12], which is too large to access the two partially blocked active sites in the compact tetramer structure. From fluorescence polarization spectroscopy measurement it was found that discrete segments of AChE tetramer might move independently [27]. The binding of bulky ligands such as fasciculin or antibodies can fix the tetramer in certain conformational states.

The presence of a spectrum of conformations for AChE tetramers further complicates the function of AChE. It has been known that the rapid opening/closing motion of aromatic residues in the peripheral anionic site of AChE functions as a gate for substrate entering the active site [28,29]. Future modeling of AChE function will have to consider these low-frequency large-scale motions as well.

## Materials and Methods

### [AChE_T_]_4_–ColQ complex model building.

The crystal structure of the [WAT]_4_PRAD complex was downloaded from the Protein Data Bank [11]. Chains A–D and I were selected for modeling. In building a complete [AChE_T_]_4_–ColQ complex model, we added the missing residues 35–40 in WAT and extended PRAD to 16 more residues in the N-terminus and 19 more residues in the C-terminus using SYBL (Tripos, St. Louis, Missouri, United States). The conformations of these residues were copied from the existing chains, i.e., the area with residues 35–40 was the same coiled α helix as the rest of the WAT, and all residues in ColQ were in polyproline II conformation. Mouse sequence of ColQ tail was downloaded from the Swiss-Prot database. While the sequence in the [WAT]_4_PRAD complex crystal structure was from human, the only differences were D35E in the WAT and T3M in the PRAD from human to mouse. SeMet21 was mutated back to Met.

The next step was to connect WAT to AChE to form AChE_T_. The mouse AChE structure was taken from a preoptimized structure based on the PDB entry (see Accession Numbers section) [29]. Since both the C-terminus of AChE catalytic domain and the N-terminus of the WAT domain have α-helical conformations, we initially connected the two using continuous α-helical conformation. A perfect α-helix sequence was used as a bridge in matching the two ends. However, the AChE had a severe clash with the C-terminal extension of PRAD in ColQ. The phi and psi angles of residues A542, T543, and D544 were manually adjusted to bend the helix to move AChE away from ColQ using known helix-bending structures in PDB. Most of the helix bending structures involve a proline residue in the kink region [30], including P410 and P537 in the mouse AChE structure itself. Here we borrowed the same helical bend from the M2 helix of the nicotinic acetylcholine receptor structure at position L11′(see Accession Numbers section) [31], which does not involve a proline residue. SYBYL version 7.0 (Tripos) was used to copy the main chain conformation of L11′ and the two adjacent residues. By geometrical matching using the 4-fold screw symmetry, we added three other chains to build a complete mouse [AChE_T_]_4_–ColQ complex model. VMD version 1.82 [32] was used to match structures in a tcl script. The complex structure was further optimized with Amber94 force field [33], using the sander module in Amber8 [34]. The initial structure had some severely bad contacts, which caused the sander minimization module in Amber8 to crash. We found that the old-style velocity quenching in NAMD v2.5 [35] worked better in removing bad clashes. The partially relaxed structure of the [AChE_T_]_4_–ColQ complex model was further optimized by 5,000 steps of conjugate-gradient energy minimization, using the sander module of Amber8 (first 100 steps were steepest descent search). The solvation effect was mimicked with a distance-dependent dielectric coefficient of 4r. A 20-Å cutoff was used in evaluating nonbond interactions. The final RMS force in energy minimization was 0.01 kcal/mol/Å.

### BNMA.

Although MD is useful in observing conformational changes in protein simulations, the size of the system prevents us from running MD for the extended time required for observing large conformational changes [15]. NMA is another useful tool to predict conformational changes from low-frequency modes [25]. Recently NMA has been extended to large biomolecules using the Elastic Network Model or BNMA [16,22,24,36]. Here we use the BNMA method to analyze the low-frequency motions of the [AChE_T_]_4_–ColQ complex model. The advantage of BNMA is that the real atomic potential can be used and projected to a smaller dimension using blocks of atoms [22]. Each residue was chosen as a block in the current study. Therefore the degrees of freedom of the system can be reduced from 3*N* to 6*M,* where *N* and *M* are the total number of atoms and residues, respectively. The factor of six in block space comes from the fact that each block has three degrees of freedom from translation and three from rotation. The rotational degrees of freedom within a block have been shown to be less important and can be omitted to further reduce the computational cost [21].

In BNMA, each component of the Hessian matrix, i.e., the second derivative of energy, was calculated and projected to the block space “on the fly” [22]. The rotational degrees of freedom for each block were omitted in this study. In this system, there are 36,836 atoms in 2,379 residues. Using BNMA reduced the total degrees of freedom from 36,836 × 3 = 11,0508 to 2,379 × 3 = 7,137. The block Hessian matrix was diagonalized by using the nmode module of Amber8. Due to the use of cutoff for nonbond interactions, the matrix is sparse and easy to solve. The whole procedure took about 2 h in a Pentium 2.8 GHz Linux machine.

From the BNMA results, RMSF of each residue can be calculated by summing over contributions from all the normal modes [37]:


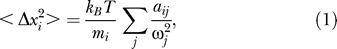


where *k_B_* is the Boltzmann constant, *T* is the temperature, *m_i_* is the mass for residue *i, a_ij_* is the *i*-th component in the eigenvector corresponding to mode *j,* and ω*_j_* is the angular frequency for mode *j.* The summation is over all the nontrivial normal modes.

The correlation map defines the correlation of motions of each residue pair and can be computed as follows [37]:


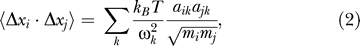


and


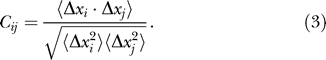


A conformational change can be considered as the linear combination of motions from all the normal modes. Given a conformational change, the involvement coefficient of mode *k* can be calculated as follows [22],


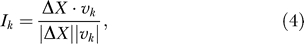


where *v_k_* is the eigenvector corresponding to mode *k,* and Δ*X* is the conformational change vector, which can be computed as the difference in coordinates of two different conformations. A large involvement coefficient indicates that the motion along this normal mode is highly relevant to the conformational change being examined. The cumulative involvement for the first *n* normal modes can be obtained by [22]


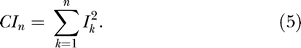


Obviously, if the summation is over all the modes (3*N*), the cumulative involvement is expected to be one.

## Supporting Information

### Accession Numbers

The accession numbers for the proteins described in this paper can be found in the SwissProt database (http://www.ebi.ac.uk/swissprot): AChE (P21836), ColQ (O35348), and PRiMA (Q810F0); and in the Protein Data Bank (http://www.rcsb.org/pdb/): PRAD/WAT complex (1VZJ), compact AChE tetramer structure (1C2O), loose AChE tetramer structure (1C2B), [WAT]_4_PRAD complex (1VZJ), mouse AChE (1MAH), and acetylcholine receptor structure (1OED).

## Abbreviations

AChE: acetylcholinesterase
AChE_T_: acetylcholinesterase T-subtype
BNMA: block normal mode analysis
ColQ: collagen-like Q subunit
MD: molecular dynamics
NMA: normal mode analysis
PRAD: proline-rich attachment domain
PRiMA: proline-rich membrane-anchoring protein
RMSD: root mean square deviation
RMSF: root mean square fluctuation
WAT: tryptophan amphiphilic tetramerization
